# Supplementary material for: Genetic variants associated with longevity in long-living Indians
Source: NPJ Aging. 2024 Nov 20;10(1):51. doi: 10.1038/s41514-024-00179-9 (PMC11579347; doi:10.1038/s41514-024-00179-9)
Supplement: Supplementary file 1 — Supplementary Information [file 41514_2024_179_MOESM1_ESM.pdf]

Supplementary Information

Custom Chip Development

Variants or Single nucleotide polymorphisms (SNPs) associated with various disorders in major therapeutic areas, such as oncology, gastroenterology, endocrinology, neurology, autoimmune and cardiovascular were obtained from GWAS Catalog [1], SNPedia [2], Clinvar [3] and through literature mining. In the case of SNPs retrieved from GWAS Catalog, p-value threshold was fixed at 0.0001. In the case of SNPs retrieved from SNPedia, magnitude of association was fixed at 1.1. Pathogenic, likely pathogenic and risk factor variants were retrieved from Clinvar. This initial screening resulted in ~500,000 variants. Further screening was done to retain variants that came from at least two sources, and have at least one publication either in NCBI Pubmed or Google Scholar. Pharmacogenomic associations were obtained from US Food and Drug Administration (FDA) Table of Pharmacogenetic Associations [4] and Clinical Pharmacogenetics Implementation Consortium (CPIC) guidelines [5]. To make the chip more comprehensive and interesting for genetic testing, ~500 variants associated with vitamin deficiencies and traits such as skin color, hair texture, adaptation to higher altitudes etc. were hand-picked from GWAS catalog, and added to the list. As the objective was to develop a low-cost chip, final selection was restricted to ~10,000 variants. Probe design was done using Illumina's DesignStudio [6], a web-based tool that helps in designing and ordering custom genotyping arrays. The tool designed unique single probes for bi-allelic variants, but designed multiple probes for multi-allelic variants and variants with low likelihood of genotyping success. 10,133 probes corresponding to 8,768 variants passed all the quality metrics required for probe design. Using the 'score' file given by DesignStudio, and working together with Illumina, Infinium iSelect HTS Custom Genotyping BeadChip [Catalog ID: WG-405-1014] was developed that can accommodate 24 samples on a single BeadChip.

Principal component analysis (PCA)

Genotype data of study samples was combined with that of 2,504 samples of known ethnicity from 1000 Genomes Project [6]. Out of 5,336 variants retained after pre-processing on study data, 5,281 variants were common across both the datasets. Principal component analysis (PCA) was done on the combined dataset of 3,771 samples using PLINK 1.9 software. Figure S1 shows the plot of top two principal components, where South Asian (SAS) samples and study samples were highlighted in different colors. Separate PCA plots were given for study samples to visualize the genetic affinity between cases and controls.

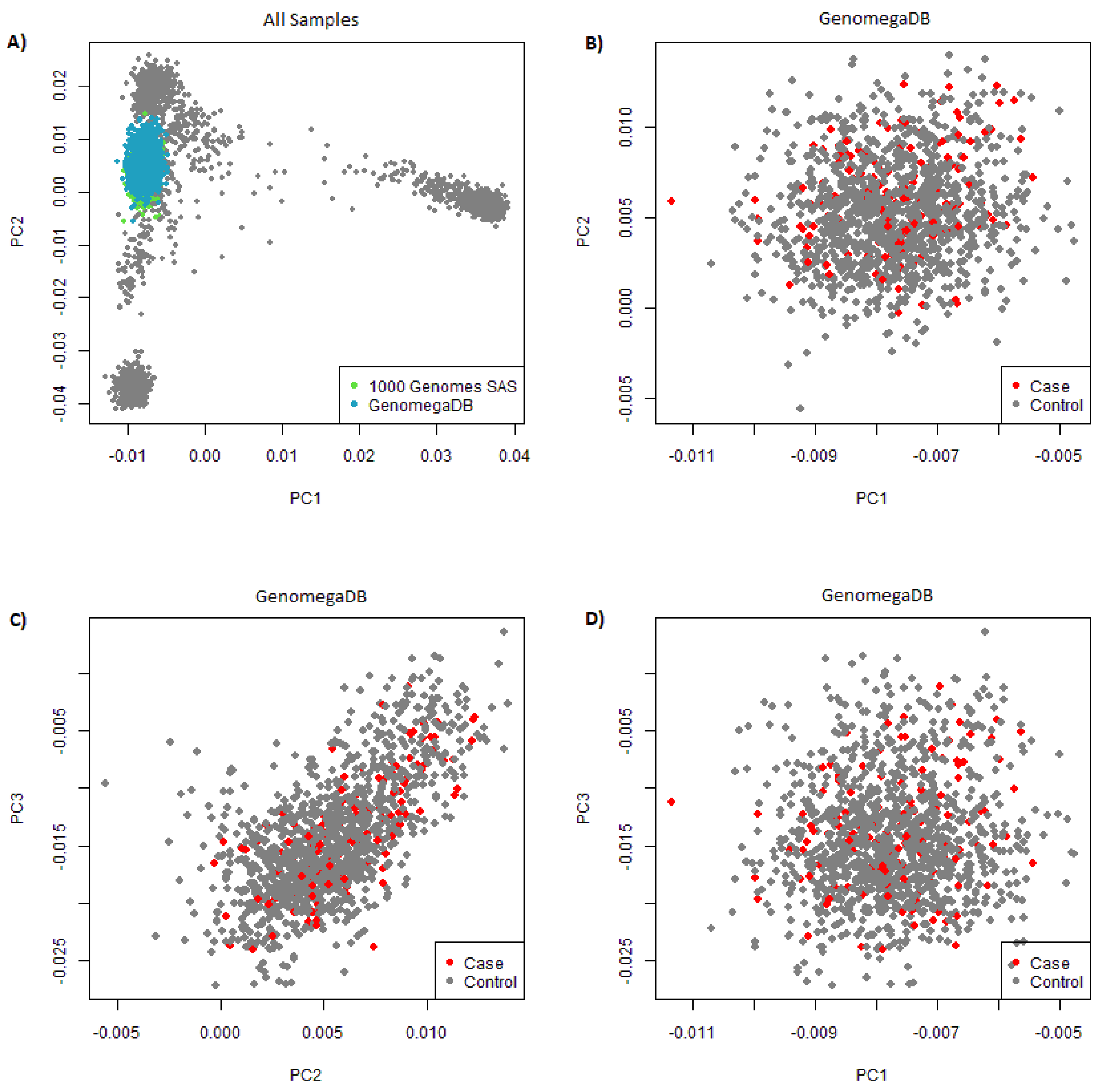

Figure S1 Principal component plots of samples from 1000 Genomes Project and GenomegaDB

A) 2,504 samples of known ethnicity from 1000 Genomes Project and 1,267 Indian samples from GenomegaDB B), C) and D) Samples of GenomegaDB on pairs of first, second and third principal components

Quantile-Quantile (Q-Q) Plot

Q-Q plot of association was obtained to evaluate the observed and expected distributions of p-values (Figure S2). *CMplot* function available in *CMplot* package of R4.3 was used to generate the plot. The deviation observed in the Q-Q plot could be due to smaller set of SNPs used in the study, in contrast to the larger set of SNPs generally used in genome-wide association studies.

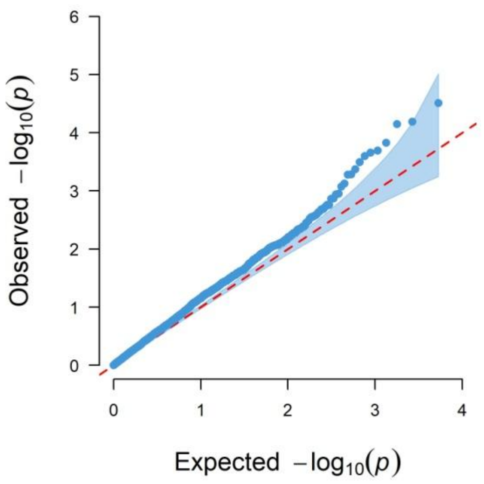

Figure S2 Q-Q Plot of association analysis

X-axis shows the negative logarithm of expected p-value. Y-axis shows the negative logarithm of observed p-value in logistic regression. Blue dots are the SNPs. Diagonal line represents expected values under hypothesis of no association.

REFERENCES

1. Sollis E, Mosaku A, Abid A, et al. The NHGRI-EBI GWAS Catalog: knowledgebase and deposition resource. Nucleic Acids Res. 2023;51(D1):D977-D985. doi:10.1093/nar/gkac1010
2. Cariaso M, Lennon G. SNPedia: a wiki supporting personal genome annotation, interpretation and analysis. Nucleic Acids Res. 2012;40(Database issue):D1308-D1312. doi:10.1093/nar/gkr798
3. Landrum MJ, Lee JM, Riley GR, et al. ClinVar: public archive of relationships among sequence variation and human phenotype. Nucleic Acids Res. 2014;42(Database issue):D980-D985. doi:10.1093/nar/gkt1113
4. US Food and Drug Administration. Drugs@FDA: https://www.fda.gov/medical-devices/precision-medicine/table-pharmacogenetic-associations
5. Clinical Pharmacogenetics Implementation Consortium. Clinical practice guidelines: https://cpicpgx.org/
6. https://cran.r-project.org/web/packages/plinkQC/vignettes/Genomes1000.pdf
